# Evaluation of Cysteinyl Leukotriene Signaling as a Therapeutic Target for Colorectal Cancer

**DOI:** 10.3389/fcell.2016.00103

**Published:** 2016-09-21

**Authors:** Lorraine Burke, Clare T. Butler, Adrian Murphy, Bruce Moran, William M. Gallagher, Jacintha O'Sullivan, Breandán N. Kennedy

**Affiliations:** ^1^UCD School of Biomolecular and Biomedical Science, UCD Conway Institute, University College DublinDublin, Ireland; ^2^Translational Oncology, Trinity Translational Medicine Institute, Department of Surgery, Trinity College Dublin, St. James's HospitalDublin, Ireland; ^3^Department of Medical Oncology, Sidney Kimmel Comprehensive Cancer Center, Johns Hopkins HospitalBaltimore, MD, USA; ^4^Trinity Translational Medicine Institute, Department of Surgery, Trinity College Dublin, St. James's HospitalDublin, Ireland

**Keywords:** cysteinyl leukotriene, eicosanoid, colorectal cancer, tumorigenesis, hallmarks of cancer, cysteinyl leukotriene receptor antagonist

## Abstract

Colorectal cancer is the third most common cancer worldwide and is associated with significant morbidity and mortality. Current pharmacotherapy options include cytotoxic chemotherapy, anti-VEGF, and anti-EGFR targeting drugs, but these are limited by toxic side effects, limited responses and ultimately resistance. Cysteinyl leukotriene (CysLT) signaling regulates intestinal homeostasis with mounting evidence suggesting that CysLT signaling also plays a role in the pathogenesis of colorectal cancer. Therefore, CysLT signaling represents a novel target for this malignancy. This review evaluates reported links between CysLT signaling and established hallmarks of cancer in addition to its pharmacological potential as a new therapeutic target.

## Introduction

Each year, ~1.4 million new cases of colorectal cancer (CRC) are diagnosed, with almost 700,000 cancer-related deaths, making it the third most common cancer worldwide and the fourth most common cause of cancer death (Torre et al., 2015). Surgical intervention is the cornerstone of treatment but adjuvant chemotherapy and targeted therapies play significant roles in improving survival. Treatment decision is guided by clinical factors (e.g., co-morbid conditions, performance status) and pathological factors (e.g., KRAS mutation status, microsatellite stability status). 5-fluorouracil-based regimens such as FOLFOX or FOLFIRI form the backbone of cytotoxic chemotherapy in the treatment of metastatic disease but are limited by chemoresistance and toxic effects on non-neoplastic healthy tissue (Longley and Johnston, 2005; Fuchs et al., 2007).

Targeted therapies include the anti-angiogenic drug bevacizumab (Avastin®), a humanized monoclonal antibody that binds directly to vascular endothelial growth factor A (VEGF-A; Ferrara et al., 2004). Blocking VEGF inhibits tumor angiogenesis, a pathophysiological process upon which solid tumors depend for growth, survival, and metastasis (Folkman, 1971). Other targeted therapies include anti-epidermal growth factor receptor (EGFR) antibodies cetuximab and panitumumab, the major advantage of which is the availability of a validated biomarker that reliably predicts patient response—patients with activating KRAS mutations have poorer progression-free and overall survival (Martinelli et al., 2009; Yen et al., 2010). Immune checkpoint inhibition is emerging as a promising treatment strategy in microsatellite instability-high (MSI-H) colorectal tumors, a phenotype resulting from defective DNA mismatch repair and accounting for up to 15% of all CRCs (Boland and Goel, 2010; Le et al., 2015). Pembrolizumab, an anti-programmed cell death protein 1 (anti-PD-1) antibody potentiates T-cell immune responses, circumventing tumor immune evasion, and significantly prolonging progression-free survival in patients with MSI-H tumors (McDermott and Atkins, 2013; Le et al., 2015). Numerous other immunotherapies are currently being evaluated in clinical trials in combination with chemotherapy regimens and/or targeted therapies.

While CRC survival rates are improving, there is a need for more effective therapies as the survival benefit associated with targeted therapies is only ~4–5 months (McCormack and Keam, 2008; Bokemeyer et al., 2012). Many novel strategies are currently under investigation, one of which is to target cysteinyl leukotriene (CysLT) signaling. The focus of this mini-review is to evaluate the link between CysLTs and the hallmarks of cancer.

## Cysteinyl leukotrienes and their receptors

Cysteinyl leukotrienes (CysLTs) are a subfamily of eicosanoids, lipophilic signaling molecules that regulate both acute and chronic inflammation (Henderson, 1994). These potent bioactive lipids are rapidly generated *de novo* from cell membrane-associated arachidonic acid (AA), an essential polyunsaturated fatty acid in response to cell activation (Clark et al., 1991). Once mobilized to the cytosol, AA is metabolized by 5-lipoxygenase (5-LOX) in conjunction with 5-lipoxygenase-activating protein (FLAP) to yield leukotriene A_4_ (LTA_4_), which undergoes further metabolism to form leukotrienes B_4_ (LTB_4_) and C_4_ (LTC_4_) (Kanaoka and Boyce, 2004). Subsequently LTC_4_ is exported from the cell via multi-drug resistance-associated proteins 1 and 4, and metabolized to LTD_4_ and LTE_4_ (Lam et al., 1989). LTC_4_, LTD_4_, and LTE_4_, referred to collectively as CysLTs, are structurally similar but exhibit functional diversity (Laidlaw and Boyce, 2012).

**Figure 1:**
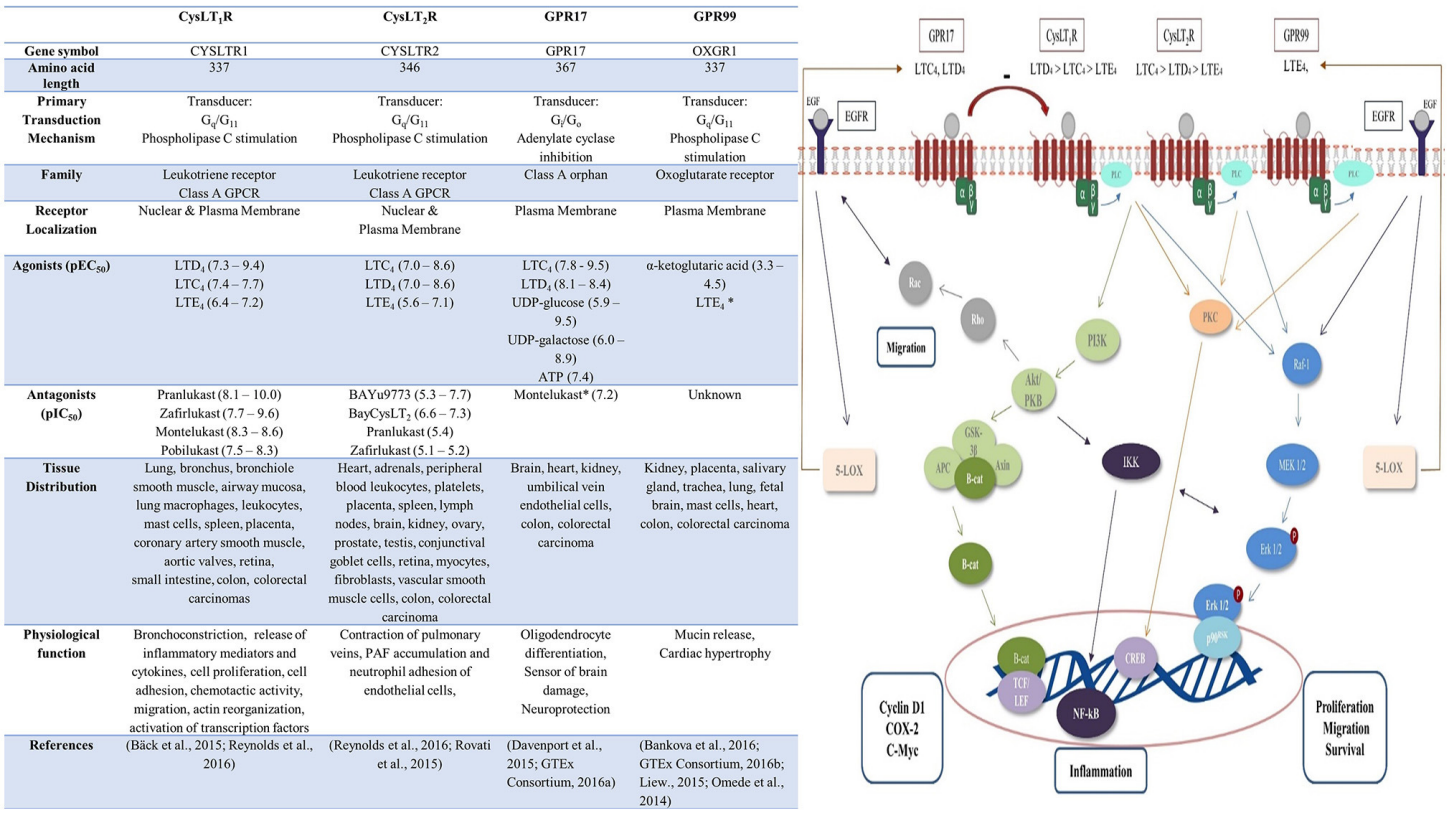
**Cysteinyl leukotriene receptors: pharmacology, distribution, function and signaling pathways**. Upon binding of ligand to the CysLT receptor various downstream signaling pathways are activated. CysLT_1_ induces PI3K-Akt signaling, resulting in β-catenin nuclear translocation and activation of target genes including cyclin D1, COX-2, and c-Myc (Savari et al., 2014). Akt/PKB activates IKK complex with subsequent degradation of IκB protein leading the NF-kB translocation and activation (Madrid et al., 2001). CysLT_1_, CysLT_2_, and GPR17 signaling can also activate phospholipase C (PLC) and Ras-Raf-MEK-ERK pathway (Thompson et al., 2008; Hennen et al., 2013; Savari et al., 2014). This lead to nuclear translocation of Erk 1/2 resulting in activation of genes involved in proliferation, migration, survival. Alternatively, CysLT receptor ligation can activate PKC and the transcription factor cAMP response element-binding protein (CREB; Savari et al., 2014). Crosstalk occurs between CysLT and EGF signaling pathways – EGF signaling also activates Rac and the Ras-Raf-MEK-ERK pathway (Magi et al., 2014). EGF signaling induces production of 5-LOX, resulting in leukotriene synthesis and consequential potentiation of CysLT receptor signaling, while LOX/CysLT_1_ also regulate EGF-induced migration (Magi et al., 2014). PLC, phospholipase C; PKC, protein kinase C; p90RSK, p90 ribosomal S6 kinase; CREB, cAMP response element binding; NF-κB, nuclear factor kappa b; IKK, IkB kinase; TCF/LEF, T-cell factor/lymphoid enhancer factor; β-cat, β-catenin; APC adenomatous polyposis coli; GSK-3β glycogen synthase kinase-3 beta; PKB protein kinase B; PI3K, phosphoinositide 3-kinase; 5-LOX, 5-lipoxygenase; EGF, epidermal growth factor; EGFR, epidermal growth factor receptor.

The biological actions of CysLTs are mediated via ligation of the widely distributed G-protein-coupled receptors, CysLT_1_ and CysLT_2_ (Figure 1; Peters-Golden et al., 2006). The role of newly-identified CysLT receptors GPR17 and GPR99 remains to be established. GPR17 is an orphan P2Y-like receptor with dual specificity for uracil nucleotides and CysLTs, while GPR99 has been proposed as a potential LTE_4_-selective CysLT receptor (Ciana et al., 2006; Kanaoka et al., 2013). Cross-regulation occurs between CysLT receptors—CysLT_2_ negatively regulates CysLT_1_ signaling via receptor heterodimerization and GPR17 has been reported as a ligand-independent negative regulator of CysLT_1_ (Lynch et al., 1999; Jiang et al., 2007; Maekawa et al., 2009).

## Cysteinyl leukotrienes and their role in cancer

CysLTs play recognized roles in promoting the inflammatory response, bronchoconstriction and vascular permeability (Davidson et al., 1987; Lee et al., 2004). CysLTs have also recently emerged as important regulators of intestinal homeostasis, with endogenous CysLT production mediating the survival and proliferation of intestinal epithelial cells (Paruchuri et al., 2006). Dysregulated CysLT signaling has been implicated in colorectal adenocarcinomas with increased CysLT_1_ and decreased CysLT_2_ levels in patient tumor samples compared with surrounding normal tissue (Magnusson et al., 2007). Interestingly, unlike the majority of G-protein coupled receptors, CysLT_1_ and CysLT_2_ are located both at the plasma membrane and the nuclear membrane (Magnusson et al., 2010). This subcellular receptor localization is critically important for CRC patient survival - patients with high nuclear CysLT_1_ expression have a poorer prognosis than patients with high cytoplasmic expression (Magnusson et al., 2010). In contrast, patients with high nuclear CysLT_2_ expression have a better overall survival expectancy, indicating the existence of an inverse relationship between nuclear CysLT_1_ and CysLT_2_ expression, and suggesting that CysLT_2_ has a protective role in CRC (Magnusson et al., 2010).

*In vitro* data corroborate these findings as malignant intestinal cell lines Caco-2 and SW480 demonstrate higher CysLT_1_ but lower CysLT_2_ expression levels compared to non-cancerous Int 407 intestinal epithelial cells (Magnusson et al., 2007). CysLT_2_ signaling also results in terminal differentiation of Caco-2 cells and growth inhibition (Magnusson et al., 2007). All-*trans* retinoic acid (ATRA), an anti-cancer agent promoting cell differentiation, acts in part by upregulating CysLT_2_ mRNA expression and LTC_4_ synthase (Bengtsson et al., 2013).

Interestingly, CysLT_1_ expression increases CRC tumor burden in a gender-specific manner *in vivo*—In the Apc^*Min*/+^ mouse model of familial adenomatous polyposis/sporadic CRC, female mice lacking the CysLT_1_ gene (*Cysltr1* ^−/−^ *Apc*^*Min*/+^) develop significantly less polyps, less systemic inflammation, and increased regulatory T-cell tumor infiltration, a negative prognostic factor in CRC, compared to *Cysltr1* ^+/+^ *Apc*^*Min*/+^ mice.

Analysis of The Cancer Genome Atlas (TCGA) RNA-sequencing dataset reveal that GPR17 and GPR99 are expressed in CRC patient samples, with GPR99 more highly expressed relative to GPR17 based on FPKM relative expression values. Kaplan-Meier survival analysis was conducted using a log-rank test with the “survival” package in R and grouping 41 tumor samples based on median expression. We found no significant correlation of either gene with overall survival. While both receptors have been identified in the colon, further investigation in a larger cohort of CRC patients is required to confirm these findings.

**Figure 2:**
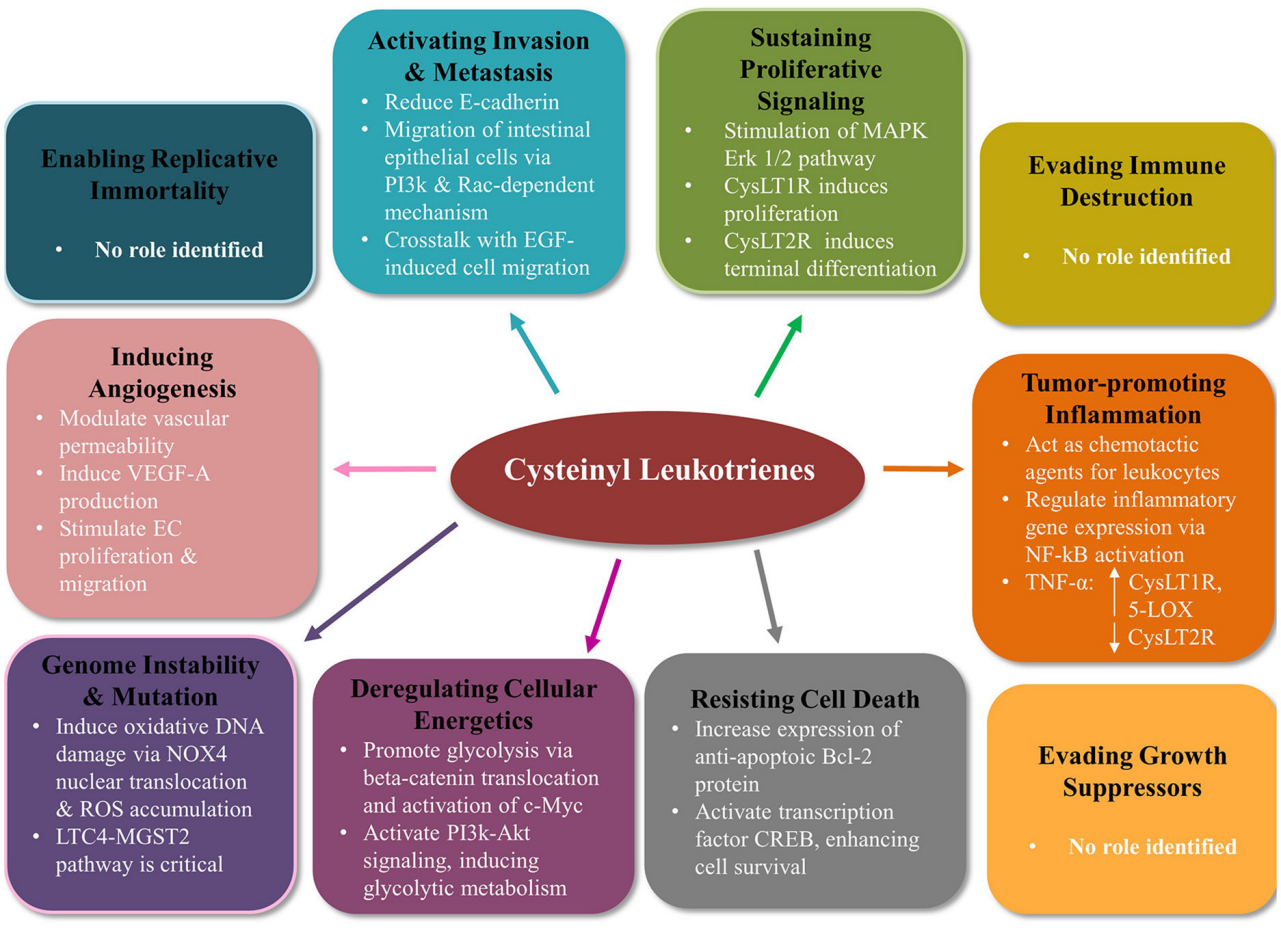
**Cysteinyl leukotriene signaling modulates many of the hallmarks of cancer in colorectal cancer**. Mounting evidence indicates CysLTs play an important role in the tumorigenesis of colorectal cancer. CysLT signaling modifies 7 out of the 10 Hanahan and Weinberg hallmarks of cancer (Hanahan and Weinberg, 2011). CysLTs sustain proliferative signaling, activate migration and invasion, induce angiogenesis, contribute to genome instability and mutation, deregulate cellular energetics, and resist cell death. No evidence to date supports a role for CysLT in enabling replicative immortality, evading immune destruction or evading growth suppressors. PI3K, phosphoinositide 3-kinase; EGF, epidermal growth factor; MAPK, mitogen-activated protein kinase; NF-κB, nuclear factor kappa b; TNFα, tumor necrosis factor alpha; CREB, cAMP response element binding; NOX4, NADPH oxidase 4; ROS, reactive oxygen species; MGST2, microsomal glutathione S-transferase 2; EC, endothelial cell.

CysLTs have multiple roles in many hallmarks of cancer (Figure 2).

### Sustained proliferative signaling

CysLTs induce cell proliferation in various cell types including bone marrow cells, smooth muscle cells, endothelial cells, and intestinal cells and it is suggested that dysregulated CysLT signaling contributes to uncontrolled proliferation (Lindgren et al., 1993; Porreca et al., 1996; Paruchuri and Sjölander, 2003; Duah et al., 2013). LTD_4_ induces intestinal cell proliferation in Int 407 cells via stimulation of the Erk 1/2 pathway and subsequent activation of p90 ribosomal S6 kinase (p90^RSK^; Paruchuri et al., 2002). LTD_4_ also reduces G_0_/G_1_ and increases S+G_2_/M phases of Int 407 cell cycle, further supporting a role in intestinal cell proliferation. CysLT_1_ signaling via the GSK-3β/β-catenin pathway increases transcription of target genes COX-2, c-Myc and Cyclin D1, well-known modulators of cell proliferation (Savari et al., 2014). The β-catenin pathway is negatively regulated by the APC tumor-suppressor protein and is critical in colorectal carcinogenesis—over 80% of sporadic CRCs carry a somatic mutation of the APC gene, while a germline mutation carries an almost 100% lifetime risk of CRC (Kinzler and Vogelstein, 1996; Jasperson et al., 2010).

The proliferative effects of CysLTs in the intestine depend on the receptor subtype. CysLT_1_ signaling induces proliferation of Int 407 epithelial cells in an autocrine manner, whereas CysLT_2_ signaling promotes terminal cell differentiation of Caco-2 cells, inhibiting growth (Paruchuri et al., 2002; Magnusson et al., 2007). CysLT_2_ expression is significantly higher during the quiescent G_0_ phase of the intestinal cell cycle compared to actively proliferating phases (Magnusson et al., 2007). In HCT-116 colorectal xenograft mouse models, CysLT_1_ antagonists reduce proliferation as determined by Ki-67 levels and significantly reduce tumor size (Savari et al., 2013). The role of non-classical CysLT receptors in cell proliferation is unknown—GPR17+ cells demonstrate proliferative activity in the central nervous system but have not been investigated in a CRC context (Ceruti et al., 2009).

### Migration, invasion

Cell migration, facilitated by the epithelial-mesenchymal transition (EMT) process, is essential for tumor invasion, angiogenesis, and metastasis. CysLT_1_ signaling stimulates the migration of Int 407 intestinal epithelial cells via a phosphatidylinositol 3-kinase and Rac-dependent mechanism (Paruchuri et al., 2005). LTD_4_ also stimulates HCT-116 colon cancer cell migration and decreases expression of adhesion molecule E-cadherin, a key mediator of the EMT process (Hirohashi, 1998; Salim et al., 2014).

Crosstalk between EGFR signaling and CysLT_1_ signaling is essential for epithelial cancer migration and invasion—the LOX/LTC_4_/CysLT_1_ signaling pathway regulates EGF-induced cell migration via Rac1 activation in A431 human epidermoid carcinoma cells (Magi et al., 2014). Conversely CysLT_2_ signaling has been shown to reduce cell migration in MCF-7 breast cancer cell lines, further substantiating many reports that CysLT receptors have opposing effects (Jiang et al., 2007; Magnusson et al., 2007, 2011a; Bengtsson et al., 2013). GPR17 signaling stimulates migration in oligodendrocytes and cardiac stromal cells but its effect on intestinal cells have not been investigated (Coppi et al., 2013; Cosentino et al., 2014).

### Angiogenesis

In order to sustain growth, a tumor must obtain a blood supply by tilting the balance of angiogenic mediators in favor of angiogenesis (Bergers and Benjamin, 2003). CysLT_1_ signaling activates production of the pro-angiogenic mediator VEGF-A *in vitro*, while CysLT antagonists modulate vascular permeability and reduce VEGF levels in murine models of allergic asthma and in asthmatic patients (Kanazawa et al., 2004; Lee et al., 2004; Poulin et al., 2011).

CysLT_1_ and CysLT_2_ receptors are both expressed in vascular endothelial cells (ECs) and vascular smooth muscle cells, either constitutively or inducibly—although CysLT_2_ is the dominant receptor type (Gronert et al., 2001; Sjöström et al., 2003; Kaetsu et al., 2007). Transgenic mice overexpressing endothelial CysLT_2_ exhibit vascular hyperpermeability and upregulate ICAM-1 and VCAM-1 expression, adhesion molecules which regulate angiogenesis (Jiang et al., 2008; Ni et al., 2014). Conflicting reports surround the regulation of EC function by CysLTs—CysLT stimulation has been shown to increase human umbilical vein endothelial cell proliferation *in vitro* (Duah et al., 2013). Yuan et al. (2009) demonstrated no proliferative effect in EA.hy926 macrovascular endothelial cells upon CysLT stimulation, solely induction of endothelial cell migration. *In vivo*, CysLTs significantly enhance microvessel growth in rat thoracic aortic ring and chick chorioallantoic membrane assays in a dose-dependent manner, an effect abrogated by both CysLT_1_ and CysLT_2_ antagonists (Tsopanoglou et al., 1994; Xu et al., 2010).

Furthermore, GPR99 and its ligand α-ketoglutarate upregulate EC proliferation and promote vessel sprouting and neovascularization using *in vivo* models of retinal angiogenesis (Sirinyan et al., 2007). GPR99 also mediates cutaneous vascular permeability in mouse respiratory models (Bankova et al., 2016).

### Oxidative DNA damage, genome instability, and mutation

DNA damage and genomic instability is a primary driver of carcinogenesis (Shen, 2011). Recent evidence highlights the role of LTC_4_ in promoting oxidative DNA damage, which if not sufficiently repaired may contribute to genomic instability and increased mutation rates (Dvash et al., 2015). A previously unrecognized microsomal glutathione-S-transferase 2 (MGST2)-LTC_4_ signaling pathway elicits nuclear translocation of NADPH oxidase 4 (NOX4) and generation of reactive oxygen species (ROS) when triggered by endoplasmic reticulum (ER) stress and chemotherapy (Dvash et al., 2015). Activation of this pathway upregulates MGST2, CysLT production enzymes and CysLT_1_ and CysLT_2_ expression in human amniotic WISH epithelial cells. LTC_4_ antagonists reduce NOX4 levels, inhibit ROS accumulation and significantly attenuate nuclear DNA damage induced by the chemotherapeutic agent doxorubicin (Dvash et al., 2015). Therefore, LTC_4_ antagonism may have potential in combination with chemotherapy to reduce toxicities.

Cancer cells often exhibit ER stress due to high proliferation rates (Wang et al., 2010). Therefore, while this pathway may not be responsible for the initiation of tumorigenesis, it may contribute to tumor progression via an ER-stress triggered mechanism of leukotriene production.

### Resisting apoptosis

CysLT signaling may induce intestinal cell resistance to apoptosis, as evidenced by the increase in cell survival of Int 407 intestinal cells exposed to LTD_4_ (Ohd et al., 2000). CysLT_1_ signaling increases COX-2 expression and subsequent expression of the anti-apoptotic protein Bcl-2 by activating the MEK/Erk signaling pathway in the Caco-2 cell line (Wikström et al., 2003). LTD_4_ promotes mitochondrial translocation of β-catenin in Int 407 intestinal cells where it induces Bcl-2 expression, thereby protecting cells from apoptosis and enhancing cell survival (Mezhybovska et al., 2006). The mechanism by which the CysLT_1_ signaling exerts this pro-survival effect has been attributed to cAMP response element-binding protein (CREB), a transcription factor implicated in the pathophysiology of a number of cancers (Paruchuri and Sjölander, 2003; Sakamoto and Frank, 2009). While CysLT_1_ signaling can inhibit intestinal cell apoptosis, CysLT_2_ signaling appears to have no effect on the levels of apoptosis in a MCF-7 breast cancer cell line (Magnusson et al., 2011b).

### Deregulating cellular energetics

The glycolytic metabolic switch adapted by tumor cells is necessary to support the demands of rapid cell proliferation, and is critically mediated by the “master regulator” c-Myc oncogene (Miller et al., 2012). CysLT_1_ signaling activates transcription of c-Myc via induction of β-catenin nuclear translocation in Int 407 intestinal epithelial cells (He et al., 1998; Ohd et al., 2000). CysLT_1_ activation also induces the PI3K-Akt signaling pathway in Int 407 cells, constitutive activation of which stimulates glycolysis (Elstrom et al., 2004; Mezhybovska et al., 2005). LTD_4_ stimulation increases metabolic activity in both non-transformed Int 407 epithelial cells and Caco-2 colon cancer cells with observed increases in ATP/ADP ratios. LTD_4_ also triggers significant increases in mitochondrial gene activity, changes in which have been identified in primary tumors of CRC patients (Polyak et al., 1998; Mezhybovska et al., 2009).

Interestingly, a key citric acid cycle intermediate α -ketoglutarate has been identified as a ligand GPR99 (He et al., 2004). α-ketoglutarate is also a substrate for prolyl hydroxylases which regulate hypoxia inducible factor 1α, a key player in the reprogramming of cancer metabolism (MacKenzie et al., 2007).

### Tumor-promoting inflammation

Chronic inflammation is a risk factor for the development of cancer, as illustrated in inflammatory bowel disease (IBD) patients (Bernstein et al., 2001). IBD patients exhibit a 3 to seven-fold higher expression of leukotriene pathway enzymes—it is currently unknown if this correlates with an increased risk of cancer development (Jupp et al., 2007). LTD_4_ antagonists reduce colonic inflammation in a rat model of acute colitis induced by intracolonic administration of trinitrobenzene sulfonic acid and may have potential in the prevention of inflammation-associated CRC (Nishikawa et al., 1995).

CysLTs act as leukocyte chemoattractants—CysLT_1_ mediates Th17 cell migration, accumulation of which correlates with the progression of inflammation-associated cancer (Kim and Lee, 2015). Tumor-associated macrophages (TAMs) within the tumor microenvironment also secrete high levels of LTD_4_ which may further promote immune cell infiltration (Zhang, 2013)_._ Tumor necrosis factor-α (TNF-α), a pro-inflammatory cytokine involved in the initiation and propagation of CRC, upregulates 5-LOX, LTC_4_ synthase and CysLT_1_ while downregulating CysLT_2_ expression in SW-480, HCT-116, HT-29, and Caco-2 colon cancer cells (Yudina et al., 2008). CysLTs also activate NF-κB, a transcriptional regulator of numerous inflammatory genes, constitutively active in up to 80% of colorectal tumors (Lind et al., 2001; Kawano et al., 2003; Hashimoto et al., 2009).

GPR17 negatively regulates CysLT_1_-mediated immune cell accumulation in the lungs, while GPR99 is proposed to elicit a pro-inflammatory response upon binding of LTE_4_, the predominant CysLT in inflamed tissue (Kanaoka et al., 2013; Akiko Maekawa et al., 2016). Autocrine CysLT_1_ signaling loops could allow intestinal epithelial cells to maintain chronic inflammation, thereby increasing the risk of inflammation-associated CRC (Paruchuri et al., 2006).

## Pharmacological potential of CysLT signaling modulators

Overall, targeting CysLT signaling is a promising option for anti-cancer therapy due to its effects on multiple oncogenic pathways described above (Figure 2). Commercially available anti-asthmatic drugs which target the CysLT_1_, have demonstrated notable *in vivo* potential as anti-cancer agents. Montelukast, a CysLT_1_-selective antagonist significantly reduces tumor volume in a HCT-116 CRC-specific murine xenograft model, via a combination of anti-proliferative and pro-apoptotic effects (Savari et al., 2013). CysLT_1_ antagonism also exhibits an anti-angiogenic effect, with reduced blood vessel formation and VEGF expression levels in colorectal tumors (Savari et al., 2013). Furthermore, pre-treatment of HCT-116 cells with montelukast prior to inoculation completely inhibited tumor initiation in BALB/c nu/nu mice. Montelukast also has antagonistic actions at GPR17—it is unknown if this receptor is important for the drug's anti-neoplastic effect (Ciana et al., 2006). Pranlukast significantly attenuates chemotherapy-triggered morbidity in mice via a MGST2-LTC_4_ pathway, and so may have therapeutic potential in combination with chemotherapy to reduce toxic side effects or to reduce the risk of metastases (Dvash et al., 2015). Furthermore, CysLT_1_ antagonists potently inhibit the growth of HCT-116 colon cancer cells, in addition to prostate, urothelial, and neuroblastoma cancer cell lines (Matsuyama et al., 2007, 2009; Sveinbjörnsson et al., 2008; Savari et al., 2013).

No studies to date have investigated the effects of specific CysLT_2_ agonists or antagonists on tumor progression specifically. In the case of CRC, selective CysLT_2_ agonism would appear to be the desired approach, but this needs potent selectivity in order to avoid negative effects of CysLT_1_ agonism on the intestine. Due to cross regulation between CysLT receptors, such a targeting strategy would need to be approached with caution (Laidlaw and Boyce, 2012).

Targeting 5-LOX or FLAP to inhibit endogenous production of CysLTs is an alternative strategy that would circumvent the increasing number of CysLT isoreceptors. Zileuton, a 5-LOX inhibitor, prevents colonic polyp formation in an APC^Δ468^ mouse model of polyposis and significantly decreases tumor burden in LoVo and HT29 colon cancer murine xenograft models (Melstrom et al., 2008; Gounaris et al., 2015). MK-866, a FLAP inhibitor, reduces proliferation of Caco-2 and HT29 colon cancer cell lines (Ford-Hutchinson, 1991; Cianchi et al., 2006).

## Conclusion

These studies highlight the importance of CysLT signaling in intestinal biology, and its potential role in the tumorigenesis of colorectal adenocarcinoma. CysLT signaling is linked with many hallmarks of cancer—cell proliferation and survival, cell migration, angiogenesis, genomic instability, glycolytic metabolic switch, and tumor-promoting inflammation. Data largely centers on CysLT_1_ and CysLT_2_ with further investigation necessary to establish the importance of GPR17 and GPR99 in this context.

Targeting CysLT signaling is a promising pharmacological strategy, as evidenced by the numerous CysLT antagonists which have anti-tumor efficacy in *in vitro* and *in vivo* CRC models. In particular, blockade of LTC_4_ has recently emerged as a promising new therapeutic opportunity in limiting toxicity to current chemotherapy drugs. A notable advantage of many CysLT receptor antagonists is their favorable safety profile. CysLT signaling modulators may have potential to synergize with other targeted therapies to benefit survival rates. In summary, CysLT signaling is gaining increasing recognition as a mediator of colorectal tumorigenesis and pharmacological manipulation of this signaling pathway represents an exciting opportunity that merits further translational research work.

## Author contributions

LB conducted the literature review and wrote the paper. CB revised and edited the paper. AM revised and edited the paper. BM provided analysis of RNA sequencing dataset. WG provided analysis of RNA sequencing dataset. JO revised and edited the paper. BK revised and edited the paper.

## Funding

This work is supported by Irish Cancer Society Research Scholarship CRS13BUT.

### Conflict of interest statement

The authors declare that the research was conducted in the absence of any commercial or financial relationships that could be construed as a potential conflict of interest.
